# Supplementary material for: Improving tuberculosis diagnosis in South Africa’s private sector: The results of a pilot public-private mix intervention in eThekwini health district
Source: PLOS Glob Public Health. 2025 Apr 9;5(4):e0004233. doi: 10.1371/journal.pgph.0004233 (PMC11981140; doi:10.1371/journal.pgph.0004233)
Supplement: Attachment — Submitted filename: Connect TB_Response to reviewers_02Dec24.docx [file pgph.0004233.s002.docx]

Please see point-by-point **responses to reviewers in italics.**

| **Reviewers” comments** | **Point to point response by authors** |
| --- | --- |
| **Reviewer 1** |  |
| Well done to the authors on a very well written paper on an important topic. There is very limited published work on TB in the private sector in South Africa. So this paper will add scientific knowledge to this area of study.  I only have **2 minor issues:** | *Thank you for identifying this.* |
| 1. Table 1: Please check the table as there appear to be ad hoc numbers down the right hand side | *Thank you for pointing this out. It appears the line numbers became interspersed in Table 1. We have corrected this in the updated manuscript.* |
| 2. Limitations: I think the authors could explore more fully how the study results may be biased by the GPs who opted not to be included.COVID could have played a role as the authors point out, but COVID would have affected all GPs. What other underlying issues could also apply? e.g. disinterest in TB/thoughts that it is a disease that should be dealt with in the public sector etc. It is interesting that a high % of GPs who opted out had practices in township areas. | *This point is well noted. We have added the following under limitations (Line 369-375):*  *On the other hand, 61 GPs declined to participate due to lack of interest and 21 indicated they do not see clients with TB. GPs who chose to participate in the study may differ in systematic ways from those who did not. For instance those who are more interested in TB may have clients with higher risk factors and/or take different care approaches than those who did not participate. While we tried to minimise these biases by recruiting GPs from different practice areas, participation bias may limit the generalisability of our findings to the broader practice area.* |
| **Reviewer 2** |  |
| This is an important contribution to TB diagnosis in the private sector. The private General Practitioners (GPs) however 96% opted for treatment in the public sector due to free access and follow up. The GPs thus were involved in the initial assessment and referral and not continued care and support for those with TB. Of note is the query on HIV status of the patients with TB symptoms which is a positive result owing to the information system prompting. Specimen yield and quality were high and perhaps a comment on why this was possible - ? better training of GPs and enhanced communication with patients; rapid turn around time to testing (dedicated transport and dedicated laboratory support). Initiation of treatment timeously must be commended but may be due to the previuosly described factors. | *Thank you for the helpful comments. With regard to the 96% who opted for treatment in the public sector, we included the following in discussion (Lines 329-337):*  *Further consideration should also be given to private providers’ access to public stock of TB medications for uncomplicated TB, as has been proven beneficial elsewhere [22]. Some clients indicated a preference for continued private care; however, most changed their minds when confronted with an actual TB diagnosis and associated medical expenses, e.g. treatment and follow-up care. Many private providers are already registered as locations where people living with HIV can access antiretroviral therapy provided through the public system [23]. Expanding this model to include treatment for drug-susceptible TB is a priority for the national government [4], and uptake of our intervention suggests a willingness from the private sector to provide this support.*  *We also added the following statement to comment on why specimen quality may have been higher in the private sector (Lines 230-235):*  *GPs who took part in the study were trained on optimum sputum specimen collection techniques, the recency of which may explain improved quality. Other factors that may have resulted in better quality specimens in GP clinics may include more time to spend with each client to explain the process, faster time from collection to testing due to on-call transport,* clients with more severe symptoms being prioritised for testing compared to public clinics*, making successful sputum production more likely.*  *With regard to yield, we posited: “The higher yield compared to eThekwini’s public sector (7.9%) may be due to higher quality specimens, although it is also likely due to underscreening for TB, as one would expect a lower overall yield given the epidemiology of the setting.” (Line 235-237)* |
| A useful benchmark will be the cost benefit and cost effectiveness of the use of the private sector (noting no incentives) but costs of training, costs of additional support of infomation system, dedicated transport for specimens, dedicated laboratory testing and adherence support. | *This is a helpful comment. We discussed suggestions for improving model costs in an added recommendation section in response to reviewer 3 (see below). We have also noted this suggestion under recommendations and as a suggestion for future research as follows:*  *Analysing cost effectiveness of various iterations of our model would be a good next step to see what would be most appropriate. (Line 324-326)*  *Assessing cost benefit and cost effectiveness would also be important for consideration of wider scale-up, factoring in costs of later stage presentations that may result from a lack of intervention. (Line 378-380)* |
| The key issue of course is the involvement of 50.5% of GPs and submission of specimens by 19.5% of GPs. Overall 100 patients with TB were diagnosed and linked to care including some with drug-resistant TB who may have been missed in the public sector. Also of note is the heightened awareness of the 158 GPs and the 128 GPs that were trained as per study requirements.  Of course, it is not known how many GPs would have referred a patient with TB sysmptoms to the public sector without their involvement in the study. | *Thank you for this comment. We have added a section under limitations, as follows (Line 359-362):*  *Another limitation is that it is unknown how many of the GPs would have referred the 107 clients for TB testing in the public sector without their involvement in the study. However, previous research in the same GP population suggests that the intervention likely improved client outcomes by initiating TB testing at the first encounter [2].* |
| Reviwer 3 |  |
| **Reviewer #3:** The authors describe the evaluation of an intervention to improve the diagnosis of TB and HIV in private practices in a reaonably defined area in South Africa. This is a logical follow up to the referenced studies evaluating the effectiveness of private practioners in diagnosis of TB and HIV in sample patients (actors).  As the authors note, there is no defined population for the patients within the practices from whom 50% of invited physicians agreed to participate nor for the 39% who submitted patient sputum specimens during the study. There is no valid comparison for the number diagnosed nor the frequencly by individual physicians and the comparison with the national number and frequency is not valid. | *Thank you for this comment. Yes we agree that we did not define a sample population to make comparisons but we described the yield in the private sector and compared it with data from the eThekwini municipality based on the positive findings and the total clients screened. The private sector study adopted a case finding approach whereas the public sector adopted a case finding and screening approach. This is meant to serve as a descriptive statistic rather than a risk difference.* |
| The authors state that recruitment of physicians was ongoing during the study, but then a calendar-defined 11 month of data collection is described. Presumably some physician began the fiirst month and completed 11 months and others only a few months? If so this makes Table 1 impossible to intrepret. How many practice-months were in the study? Row percentages rather than columns would be of more interest. | *Thank you for bringing our attention to these points. We have now included a sentence in findings indicating that GPs contributed a total of 1432 practice months to the study (median 10.5 months, IQR 6-11) (Line 142-143).*  *We have also updated Table 1 with row percentages (Line 149) and now report ANOVA tests to measure difference in provider characteristics by practice area (Line 150-154) which showed consultation fees were significantly lower among township practitioners. We have postulated that “Modest monetary incentives (e.g. ZAR100) per case identified may help to improve TB testing, especially in areas with lower consultation fees*.” *(Line 222-224).* |
| The findings as presented are that some physicians who agreed to participate and accepted "training." When they were provided with an individual capable of promtly transporting the specimens to the public lab, they were able to diagnose TB and collect HIV diagnoses (only a few with a new HIV diagnosis?).  The important questions for this implementation study (reference #9 an unpublished abstract is not helpful) are:  1. What were the physicians asked to do for training and was the length of committment to "training" a barrier? | *We appreciate your detailed consideration of these missing elements. We respectfully note that reference 9 is a published abstract and have included the reference information; however, it does not go into the detail requested, and we have thus responded with additional detail as indicated under each point. We hope this sufficiently addresses your concerns.*   *We have now added the following information to the text (Line 57-67):*  *At the project outset, GPs were asked to attend two one-hour virtual training sessions on sputum specimen collection and use of the Vula application, offered on evenings and weekends to accommodate GPs’ schedules. Recordings were made available for those who could not attend. Training time was not financially compensated, but participating GPs were awarded Continuing Professional Development points. As training sessions became a barrier to active GP participation, we soon developed short-form training materials and employed research assistants to provide on-the-spot training in Vula and specimen collection at the time of GP consent.* |
| 2. Were there other reasons for not participating? | *Please see Figure 1 for reasons for GP non-participation. Additionally, we have added the following statement to limitations (Line 370-375):*  *61 GPs declined to participate due to lack of interest and 21 indicated they do not see clients with TB. GPs who chose to participate in the study may differ in systematic ways from those who did not. For instance those who are more interested in TB may have clients with higher risk factors and/or take different care approaches than those who did not participate. While we tried to minimise these biases by recruiting GPs from different practice areas, participation bias may limit the generalisability of our findings to the broader practice area.* |
| 3. Was compensation for training time provided to participants? Would that be required for a non-study implementation? | *Please see response to 1 above.* |
| 4. How was the transportation of specimens funded? If so, how would this be funded more broadly? | *We have clarified in methods that drivers were employed by the project (Line 79) and included additional considerations under discussion, as outlined in 5 below.* |
| 5. Do the authors conclude this approach warrant implementation or further study? What are the necessary components beyond providing rapid, accurate specimen testing and public health treatment access? | *We have now added recommendations to the implications section, which include the following (Line 309-344):*   - *Action is needed to improve GP awareness of TB in the private sector and update GPs on national guidance for screening and testing. This could be achieved through government-endorsed webinars (e.g. through the South African knowledge hub), engagement with Independent Practice Associations, and through development of private practice policies for TB and HIV screening and testing under NHI.* - *Private GPs should be encouraged to collect sputum specimens on-site and to enquire about HIV whenever TB is suspected to hasten appropriate testing and treatment. Our findings suggest these steps are acceptable and feasible to clients and GPs. What happens with sputum specimens will have to be determined by setting and resources. In some settings, transporting sputa to the nearest public clinic to be collected by laboratory services may be the most practical. In settings with a higher density of GPs, dedicated transport services to an NHLS laboratory may be more economical. In our study, dedicated drivers were employed to collect and deliver specimens. Outsourcing this service to transport companies and/or use of scooters for transport between clinics or to a central laboratory could improve the affordability of the model. An agreement with NHLS transport services to collect specimens upon request as part of routine services might also be considered. Analysing cost-effectiveness of various iterations of our model would be a good next step to see what would be most appropriate. We also recommend the use of the Vula application for coordinating specimen transport and prompting HIV enquiry, as Vula is already widely used in South Africa [22], and the study-developed form can be made freely available to GPs across the country.* - *Based on our experience and others in the private-public space, we further recommend the involvement of an intermediary such as non-governmental organisations or practice associations who can understand the differing needs of the private and public health environments [26].* |
